# Implementation of Motor Imagery during Specific Aerobic Training Session in Young Tennis Players

**DOI:** 10.1371/journal.pone.0143331

**Published:** 2015-11-18

**Authors:** Aymeric Guillot, Franck Di Rienzo, Vincent Pialoux, Germain Simon, Sarah Skinner, Isabelle Rogowski

**Affiliations:** 1 Institut Universitaire de France, 75000, Paris, France; 2 Université de Lyon, Université Lyon 1, Centre de Recherche et d'Innovation sur le Sport–EA 647, UFRSTAPS, Villeurbanne, France; 3 Ligue du Lyonnais de Tennis, Bron, France; Tokai University, JAPAN

## Abstract

The aim of this study was to investigate the effects of implementing motor imagery (MI) during specific tennis high intensity intermittent training (HIIT) sessions on groundstroke performance in young elite tennis players. Stroke accuracy and ball velocity of forehand and backhand drives were evaluated in ten young tennis players, immediately before and after having randomly performed two HIIT sessions. One session included MI exercises during the recovery phases, while the other included verbal encouragements for physical efforts and served as control condition. Results revealed that similar cardiac demand was observed during both sessions, while implementing MI maintained groundstroke accuracy. Embedding MI during HIIT enabled the development of physical fitness and the preservation of stroke performance. These findings bring new insight to tennis and conditioning coaches in order to fulfil the benefits of specific playing HIIT sessions, and therefore to optimise the training time.

## Introduction

Tennis players alternate high intensity actions with both passive and active recovery phases during a match [1]. A high aerobic capacity is thus required to delay fatigue during repeated sprints [2], improve speed recovery, and maintain the concentration [3]. Practically, the time allocated to enhance aerobic fitness in young tennis players remains somehow limited as they spend a large amount of time for the practice of technical and tactical drills [4]. High intensity interval training (HIIT) based on game-specific on-court drills has thus been recommended to enhance aerobic performance. Such specific session aims at maintaining the technical skills and preserving the training time [5–6]. A pioneer study however provided evidence that specific playing HIIT session might fulfil the aerobic objectives in terms of cardiac demand, but may also result in reduced groundstroke velocity and accuracy in young tennis players [7]. As such training session is crucial to optimise the training time, alternative strategies should be determined to abolish the decrease in groundstroke performance.

Imagining and simulating the sensations of an action without engaging in its physical execution is one of the most remarkable human mental capacities [8], and there is now compelling evidence that motor imagery (MI) is a reliable strategy to enhance motor performance and promote motor learning [9–11]. MI and motor execution share several parallel characteristics at the behavioural and physiological levels [12]. While the neural networks underlying individual imagery ability and the degree of motor expertise are partially distinct [13–14], the functional equivalence between MI and motor performance is also well-established [15]. Interestingly, the activity-dependant neuroplasticity elicited by MI practice is similar to that observed after physical practice of the same motor skill [16–18]. Both this behavioural matching and the high overlap of active brain regions strongly support the effectiveness of implementing MI sessions in the preparation of athletes [19].

Previous research provided evidence of beneficial effects of MI practice in both young and adult tennis players [20–25]. As MI is known to be more effective in closed skills, where the environment is predictable [22], most of these experiments focused on the tennis serve, which substantially contributes to win or gain advantage in the point. Data showed that, to be effective, MI has to be congruent with physical practice. Matching the environmental context of physical practice by performing MI in an adequate ecological situation, and replicating the sensory inputs associated with holding appropriate implement, are therefore two major key-components that should be addressed to develop effective MI programs [9,26]. An appropriate competitive environment is likely to facilitate athletes’ ability to recall and rehearse a motor sequence by providing usual and relevant feedback before imagining [27]. As well, Mizuguchi et al. [28] showed that appropriately holding a tennis racket improved the quality of MI, whereas holding a non-specific tool substantially hampered MI accuracy [29]. Nevertheless, very few experimental studies investigated the effects of an embedded MI training program where athletes perform imagery trials between sets of corresponding physical practice. Hence, looking for MI benefits on tennis groundstroke performed during a rally (i.e., forehand and backhand shots) requires direct experimental investigation.

The aim of this study was to investigate the benefits of performing MI during specific inter-trial periods of HIIT in young tennis players. As MI is known to primarily enhance the technical components of a motor skill [30], we hypothesized that MI would not affect the cardiac activity during the specific playing HIIT session which need to stay sufficiently high to induce significant improvement of aerobic performance, but might contribute to preserve the groundstroke velocity and accuracy in spite of physical fatigue elicited by HIIT.

## Materials and Methods

Four girl and six boy right-handed elite tennis players (age: 13.5 ± 0.8 years; height: 1.61 ± 0.08 m; mass: 47.2 ± 10.3 kg; tennis practice: 8.3 ± 1.8 years; weekly tennis training: 9.2 ± 1.4 hours; weekly conditioning training: 3.0 ± 0.5 hours; French tennis ranking: from 3/6 to 15; maximal shuttle velocity [31]: 12.8 ± 0.9 lm.h^-1^; maximal heart rate: 199 ± 5 beats per minute) volunteered to participate in this study, which was approved by the ethics committee Sud-Est II (IRB 00009118). Written informed parental and player’s consents were obtained before data collection. All players completed the French version of the Revised Movement Imagery Questionnaire (MIQ-R) [32–33] to evaluate visual (24 ± 2 points) and kinaesthetic MI ability (17 ± 8 points).

All players randomly performed two playing training sessions: a session including MI practice and a control session without MI but a neutral activity during equivalent time (C), separated by one week. Each session started with a 15-min standardized warm-up [34], immediately followed by an evaluation of tennis groundstroke performance. Then, each player performed either the MI or C session, before a last stroke performance evaluation.

The groundstroke performance evaluation followed exactly the procedure described by Pialoux et al. [7]. Briefly, players randomly performed one set of 10 crosscourt forehand drives, and another set of 10 crosscourt backhand drives. They were instructed to hit as fast as possible toward a target, while focusing on achieving a winning shot. A radar gun (Stalker Pro II, Stalker Radar, Plano, TX, USA) was located behind the player to record ball velocity. The tennis ball was projected by a ball machine (HighTOF, Echamboulains, France). To assess shot accuracy, a target with four areas was drawn on the court, from the baseline and the alley line, in the left corner of the opposite court for the forehand dive and in the right corner of the opposite court for the backhand strokes. A ball bounce in the area 1*1 m accounted for 1 point; in the area 2*2m for 2 points; in the area 3*3 m for 3 points; in the area 4*4m for 4 points; in the opposite court for 5 points. Another location of the ball bounce resulted in eight points. For each type of stroke, the accuracy was defined by the sum of all points, with a lower score corresponding to a higher accuracy, while stroke velocity corresponded to the mean velocity of all strokes.

The maximal shuttle velocity was used to individualise HIIT sessions [7], which consisted of two 6-min sets interspersed with 5-min recovery. The first set was composed by a series of 10 s bouts at 110% of maximal shuttle velocity and 20 s of passive recovery. The second set was composed of 15 s bouts at 105% of maximal shuttle velocity and 20 s of passive recovery. The design of the HIIT session was exactly the same as described by Pialoux et al. [7]. All players were instructed to move as fast as possible, and hit with maximal effort while concomitantly maintaining stroke accuracy. Heart rate (HR) was recorded (Polar RS800, Polar Electro, Finland) and analysed with PolarPro Trainer 5 software (Polar Electro, Finland), exercising HR data being expressed as percentage of maximal HR. HR was averaged by both 6-min sets, and the time spent in five predetermined zones of maximal HR during each set was computed: 80–85%, 85–90%, 90–95%, and 95–100%. The rating of perceived exertion [35], which examines the players’ subjective difficulty of each session, was collected immediately after MI and C sessions.

Two sessions of MI were performed during the recovery periods scheduled right after each HIIT session. Among the five min dedicated to these recovery periods, the MI intervention lasted four min. Players had to independently mentally simulate both forehand and backhand strokes. The MI session was supervised by the same experimenter and performed in the court player seating. All participants were familiarised with MI use before the experiment. During the first 30 s, the player was requested to relax and breathe deeply with slow inhalation through the nose. Then, he/she imagined 6 perfect forehand drives with the ball bouncing in the 1*1 m area (i.e., the best accuracy score). Strict MI instructions were written down and scrutinized to avoid the occurrence of experimenter bias, and to ensure that participants followed the imagery instructions (see below). The imagery script was developed focusing on both visualisation of the movement (through internal or external imagery), and feeling of the movement, force and balance (kinaesthetic imagery). Practically, players were requested to combine either internal or external visual imagery along with kinaesthetic imagery at their convenience, in order to emphasize the vividness and accuracy of the mental representation. Guidelines further included details of the movement as well as visceral and somatic responses accompanying the action. Finally, players were requested to control imagery speed by imagining the movement in real time, i.e. as if they were physically performing it. During the second part of the MI intervention, players mentally performed a total of 6 backhand strokes following same specific guidelines. Individual debriefings were scheduled at the end of the session to investigate adherence of the participants to MI instructions, and to determine whether they encountered any difficulty in forming mental images. Accordingly, they were asked to describe their ease of seeing and feeling the movement, and to report whether they used internal, external visual imagery or switched between these two imagery perspectives.

*Imagery script*: *Start by getting a comfortable position*, *and take note of how you are feeling right now*… *You might want to close your eyes or focus your gaze on one spot in the room*. *Take a deep breath in*. . . . *Filling your lungs*. . . . *And now breathe out*, *emptying your lungs completely… Breathe in again through your nose*. . . . *And blow the air out through your mouth… Keep breathing like this and allow your body to relax from any tension… Now picture yourself on the court… Slowly scan the scene… You are completely focused on your stroke and you start moving*, *just as you are moving when you prepare to return… Feel now your muscles ready to spring into action… You are ready to do everything as it should be done… Pushing yourself above and beyond your expectations… You are mentally strong and get ready for the session… See the ball arriving while you are preparing your forehand [backhand]… Feel the muscles in your leg as you move forward and in your arm as you prepare to hit the ball… Get ready to imagine the movement at the same speed as if you were physically executing it*, *concentrating on the explosive power of the movement… Feel the rotation of your hips and shoulders as power develops and is transferred up your arms and hands to the ball… You hear and feel the contact of the ball on the racket and see the ball explode off the racket… Mentally perform this motor sequence 5 more times by concentrating on each detail of the movement and feeling each muscle contraction…*

During the C sessions, players spent equivalent time in the presence of the experimenter, but did not perform any form of MI. Instead, they received positive feedback and were encouraged on the pursuit of physical effort. Practically, the experimenter encouraged the player for 4 minutes during each C session, without augmenting their interaction by considering any technical advice or any form of mental practice. Such context primarily reflects one of the conventional roles of the coaches during actual aerobic training.

All data are presented as mean ± standard error (SE; See S1 File for raw data). After verifying normality and homoscedasticity, analyses of variance (ANOVAs) with two repeated factors (aerobic training session: MI vs. C; and time: pre- vs. post-test for groundstroke performance or 1st set vs. 2nd set for HR comparisons) was applied to the physiological responses and tennis performance outcomes. When ANOVAs revealed a significant difference, p-value and partial eta squared (η^2^), and their interpretation according to Cohen’s scale (η^2^ = 0.01 for small effect, η^2^ = 0.06 for medium effect, and η^2^ = 0.14 for large effect [36]) were reported. Bonferroni post-hoc test were performed to investigate statistically significant interactions. A paired-t test was used to compare RPE between MI and C sessions. The level of significance was set at p ≤0.05. Analyses were performed using SPSS 11.0 (SPSS, Inc., Chicago, IL.).

## Results

ANOVA showed large effects of set for mean HR (η^2^ = 0.53; p = 0.02), mean percentage of HRmax (η^2^ = 0.53; p = 0.02), mean time spent at 80–85% of HRmax (η^2^ = 0.45; p = 0.04), at 85–90% (η^2^ = 0.43; p = 0.04), and at 95–100% (η^2^ = 0.64; p = 0.01), while no effects were reported for mean time spent at 90–95% of HRmax (Table 1). The mean HR and percentage of HRmax was higher during the second set compared to the first one (Table 1). The mean time spent at 80–85% and 85–90% of HRmax was lower during the second set when compared to the first one, while the mean time spent at 95–100% of HRmax was higher during the second set (Table 1). Similar RPE scores were obtained after both C and MI sessions (15.1 ± 1.4 and 15.4 ± 1.4, respectively).

**Table 1:** Mean (± standard error) heart rate (HR), percentage of maximal HR (%HRmax), and time (s) spent in the five zones of HRmax for the first and second sets of training session including motor imagery (MI) and control (C) training sessions.

	MI		C		
	1st set	2nd set	1st set	2nd set	
HR (bpm)	171 ± 2	176 ± 2	171 ± 3	176 ± 2	†
% HRmax	86.6 ± 0.9	88.3 ± 0.7	86.5 ± 1.1	88.4 ± 0.7	†
80–85% (s)	66.0 ± 22.2	26.8 ± 3.2	43.6 ± 15.0	32.0 ± 13.0	†
85–90% (s)	122.8 ± 18.2	78.8 ± 20.1	94.4 ± 17.5	61.9 ± 11.3	†
90–95% (s)	106.5 ± 39.0	154.8 ± 17.9	186.2 ± 32.3	168.7 ± 10.4	
95–100% (s)	22.4 ± 15.3	66.0 ± 25.5	20.4 ± 7.9	67.7 ± 17.2	††

Set effect with † for p≤0.05 and †† for p≤0.01

**Fig 1:**
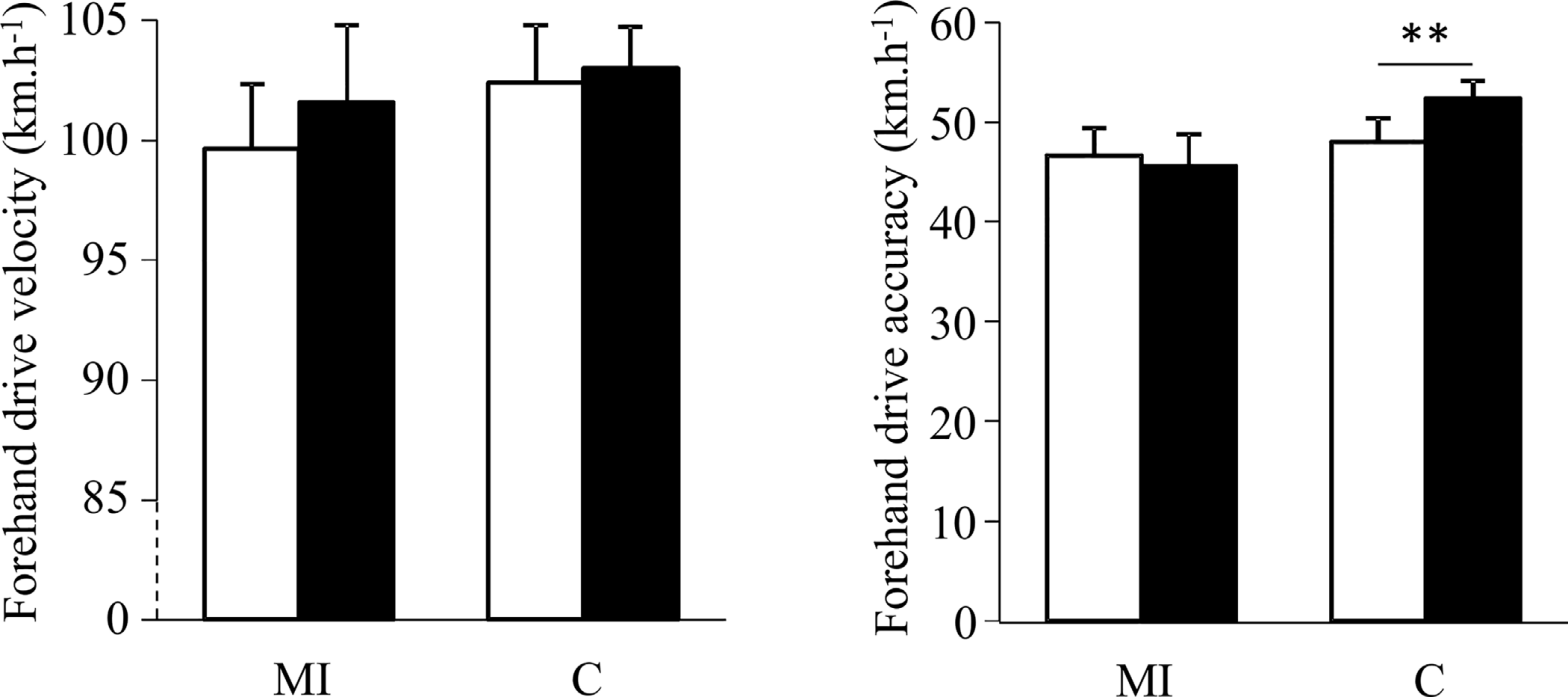
Mean (± SE) velocity (A) and accuracy (B) for forehand drive before (white) and after (black) playing aerobic session with (MI) and without (C) implementation of motor imagery; with ** for p≤0.01.

**Fig 2:**
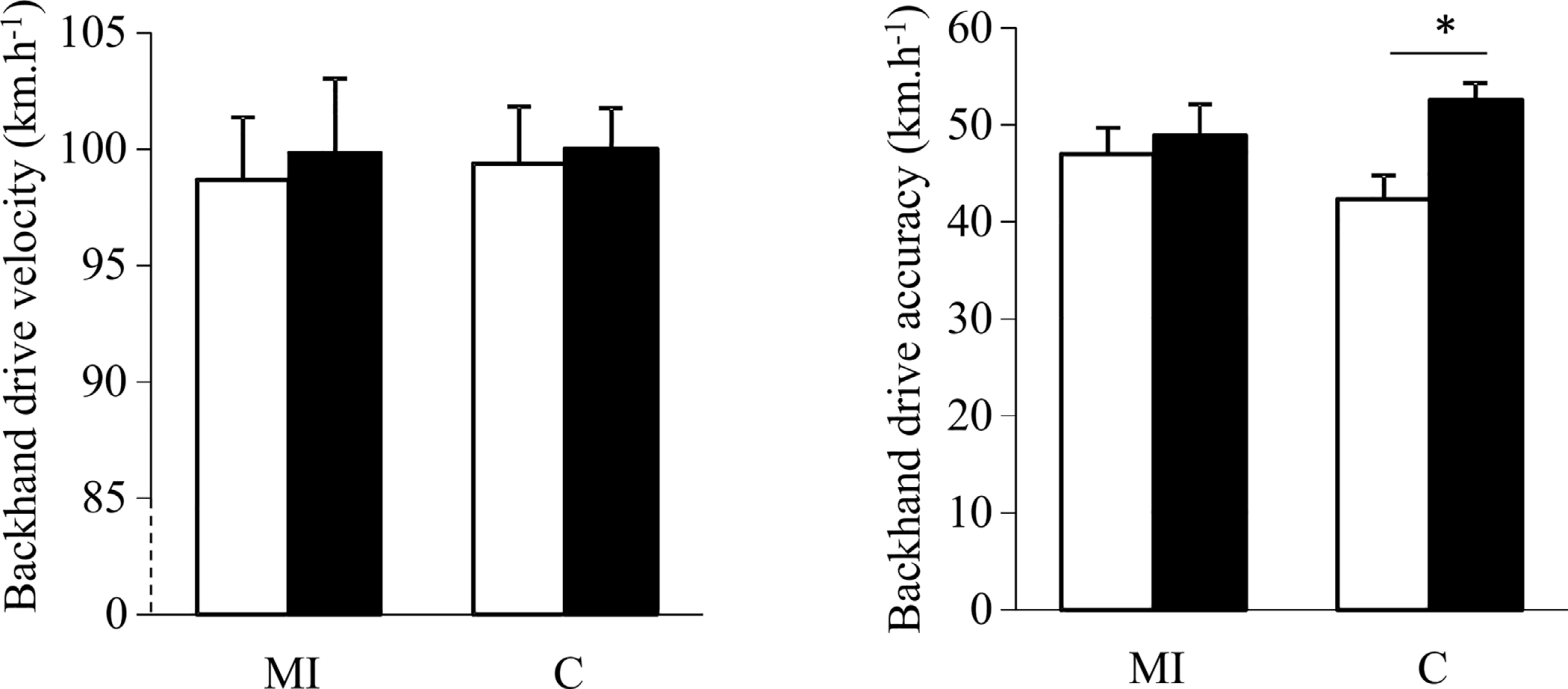
Mean (± SE) velocity (A) and accuracy (B) for backhand drive before (pre) and after (post) playing aerobic session with (MI) and without (C) implementation of motor imagery; with * for p<0.05.

ANOVA did not revealed any difference for the forehand and backhand drive velocity (Figs 1A and 2A), while a large effect of interaction between condition and time was observed for the forehand (η^2^ = 0.47; p = 0.01) and backhand (η^2^ = 0.40; p = 0.02) drive accuracy (Figs 1B and 2B). Post-hoc tests showed that groundstroke accuracy was reduced after the C session (p = 0.01 for the forehand drive score, and p = 0.02 for the backhand drive score), while no differences were found after the MI session.

When asked how concerned players were with imagining the movement, they did not report any affect or substantial difficulty to both mentally simulate the movement and perform visual and kinaesthetic imagery concurrently. All players indicated that they followed imagery instructions and that they were not concerned with imagining the movement in real time. Accordingly, they did not increase or decrease the speed of their imagery throughout the intervention. Furthermore, none of the participants reported switching from the internal to the external imagery perspective, and the great majority of the players reported performing internal imagery along with kinaesthetic imagery.

## Discussion

The present study was designed to investigate whether MI contributed to preserve the velocity and accuracy of tennis forehand and backhand strokes after HIIT based on game-specific on-court drills. The main results showed that embedded MI practice blunted the decrease of subsequent tennis shot accuracy usually observed during such HIIT training sessions [7]. Interestingly, implementing MI during HIIT did not affect the cardiac demand, hence supporting the effectiveness of performing MI in an ecological context.

It has been established that on-court training is important to improve tennis-specific performance and reduce injury [37]. The inclusion of HIIT based on game-specific on-court drills is known to concomitantly improve aerobic fitness while stimulating the tennis technical component of the game [5]. In addition, this type of training is of high interest for young tennis players, since the time dedicated to conditioning is often limited by the high amount of time practicing technical and tactical drills. However, it was previously shown that tennis skills were decreased after such training associated with HIIT [7]. As MI has been shown to enhance the technical parts of a given motor skill [30], and is a cost-effective strategy promoting motor learning, we postulated that mentally simulating subsequent efficient tennis ground strokes might contribute to reduce the detrimental effects of HIIT.

First, our data established that the cardiac demand during both MI and C sessions was similar to those reported by Pialoux et al. [7] during identical tennis HIIT session. The pattern of cardiac demand was also comparable in the two conditions, with mean HR and time spent in the high percentage ranges of HRmax higher during the second set (Table 1) suggesting a HR drift as it was reported in the previous study [7]. This difference likely results to a delayed HR kinetic and may be the sign of a higher cardiac fatigue between the first and the second set. The times spent at percentages over 90% of HRmax (Table 1) confirm that both MI and control session achieved the recommended HR required to enhance aerobic fitness [38]. This result also supports that MI did not affect the cardiac response to exercise when included within a HIIT session.

A second important finding is that performing embedded MI improved tennis performance by compensating the expected decrease in technical performance and, therefore, preserved skill efficiency. These data not only confirm the benefits of MI on tennis motor performance, but further support that implementing MI in an appropriate and ecological situation matching the context of physical practice is relevant [9–10,28,39]. Although the amount of uncertainty conveyed by the experimental situation is somewhat limited (this is not a real rally in situ), the present study highlights the benefits of a MI training program on a stroke which directly depends on the opponent’s behaviour. This finding extends previous results focusing primarily on the effects of MI on the tennis serve [20,22,24–25]. In a recent study, Gaggioli et al. [40] demonstrated the positive effect of MI on coordination in a complex motor skill involving synchronization of upper and lower limb movements. Present data support this result and promote that combined mental and physical practice within the same training session is relevant for ensuring MI efficiency, in order to vividly simulate one’s own action and integrate these predictions for coordination. Generally speaking, past experiments reported positive effects of MI practice on motor performance after long periods of training (i.e. lasting several weeks). Present data show that short term benefits on movement accuracy can be obtained in the short run when MI is embedded to the regular course of training. Physical fatigue elicited by intense aerobic practice is known to affect not only the physiological processes related to energy expenditure but also the cognitive processes underlying efficient motor control mediating movement accuracy [41]. The effects of aerobic fatigue on cognitive motor processes might account, at least to some extent, to the decrease in groundstroke accuracy usually observed during specific HIIT sessions [7]. MI practice may counterbalance such deleterious effects, as mental rehearsal is known to increase focus on movement preparation and facilitate forthcoming executions [42]. Here, we did not find any differences on groundstroke velocity. This is congruent with the assumption that MI did not affect physiological factors but rather operated on the cognitive components of the technical skills, as early underlined in pioneering reviews related to MI use in sports [43].

This study has some limitations that should be considered before drawing general conclusions. First, the sample size is somewhat limited and the study involved only young elite tennis players, hence limiting the extent and generalisability of these findings to other samples of athletes. Second, the imagery ability might have been ideally assessed using the MIQ-3 [44], which includes scales measuring internal, external, and kinaesthetic imagery, but there is not a validated French version of this instrument. Future research might explore in greater details the influence of the individual internal/external visual imagery preference, and further include a respective manipulation check. Another main limitation is that the study focused on technical skills and therefore investigated only the effects of cognitive specific MI. Based on a robust approach of the MI experience [45], future studies might consider other cognitive and motivational image content effects, by examining not only what is imaged, but also why it is imaged and how image is interpreted [45]. As well, investigating the meta-imagery processes, i.e. the athletes’ knowledge of their own imagery processes, might be relevant to evaluate MI use and efficiency [46]. Finally, this experiment provided evidence of the immediate benefits of a MI training session. Although similar positive effects following a long-term MI training program might be expected, this has not yet been tested experimentally. Testing the effects of various embedded MI content during HIIT sessions would be an exciting focus of research in the future studies.

In conclusion, the results showed a similar cardiac demand during the playing HIIT sessions with and without MI, while only the session which included MI preserved the groundstroke accuracy after training session. The findings of the current study provide evidence that implementing MI during such task-specific aerobic training allow the fulfilment of the development of physical fitness while maintaining the stroke performance. This new insight may help tennis and conditioning coaches to optimise the training time.

## Supporting Information

S1 File Raw data. (XLSX)
